# Development and Validation of a Multicyclic Peptide Targeting PD-L1 for Radiotheranostics

**DOI:** 10.1021/acsmedchemlett.5c00770

**Published:** 2026-02-16

**Authors:** Lingxin Meng, Xiaoyan Li, Jimmy S. Patel, Steven H. Liang

**Affiliations:** † Department of Radiology and Imaging Sciences, 1371 Emory University, 1364 Clifton Road, Atlanta, Georgia 30322, United States; ‡ Department of Radiation Oncology, Winship Cancer Institute of Emory University, Atlanta, Georgia 30322, United States; § Wallace H. Coulter Department of Biomedical Engineering, Georgia Institute of Technology and Emory University, Atlanta, Georgia 30332, United States

**Keywords:** Programmed death-ligand 1 (PD-L1), Disulfide-directed multicyclic peptide (DDMP), Positron emission tomography (PET), Radiotheranostics, Cancer

## Abstract

The advent of immune checkpoint blockade therapy, exemplified by inhibitors targeting programmed cell death protein 1/programmed death-ligand 1 (PD-1/PD-L1) axis, has revolutionized the landscape of clinical oncology. Despite its remarkable success, therapeutic benefits remain limited to a subset of patients, highlighting the urgent need for more accurate methods of patient stratification. Conventional techniques for assessing PD-L1 expression, such as immunohistochemistry, provide static and localized information but lack the ability to capture whole-body distribution or temporal dynamics. In contrast, positron emission tomography (PET) offers a noninvasive approach for visualizing PD-L1 expression and disease burden *in vivo*. However, clinical translation of PD-L1-specific radiotracers has been hampered by suboptimal tumor accumulation and unfavorable pharmacokinetics. To address this limitation, a recent study established a disulfide-directed multicyclic peptide (DDMP) platform capable of generating high-affinity peptide ligands specifically designed for PD-L1 imaging and potential therapeutic applications.

## Graphical abstract

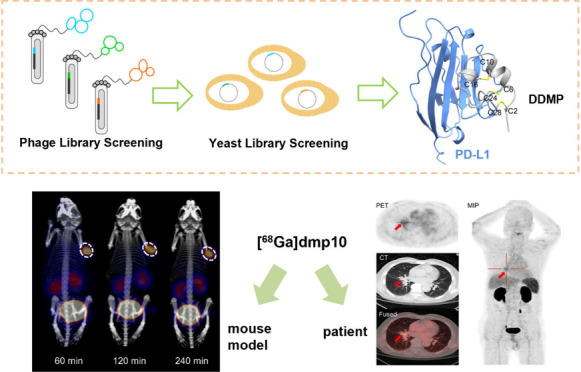

Immune checkpoint blockade (ICB) therapy promotes antitumor immunity by reversing T-cell exhaustion caused by inhibitory signaling pathways such as programmed cell death protein 1/programmed death-ligand 1 (PD-1/PD-L1).^1^ The PD-1 receptor is broadly expressed on T cells, B cells, and myeloid cells, while its ligand PD-L1, is predominantly expressed on antigen-presenting cells and tumor cells.^2,3^ Tumors exploit the PD-1/PD-L1 interaction to evade immune surveillance. Blockade of this interaction restores T cell effector function and promotes tumor regression. Nevertheless, only a subset of patients derive durable clinical benefit from PD-1/PD-L1 inhibition, highlighting the need for PD-L1/PD-1–targeted radiotracers to guide patient selection and the urgency of developing more effective therapeutic strategies.^4–6^ Current assessment of immune checkpoint expression primarily relies on immunohistochemistry (IHC), yet its utility is limited by the spatial and temporal heterogeneity of the tumor microenvironment.^7,8^ Positron emission tomography (PET) provides a noninvasive imaging modality that enables quantitative, dynamic visualization of immune checkpoint expression *in vivo*.^9–12^ Several classes of PD-L1-targeted radiotracers have been developed in recent years, including full-length antibodies, single-domain nanobodies, small molecules, and peptides, among others.^13–16^ However, radiolabeled anti-PD-L1 antibodies suffer from its unfavorable pharmacokinetics, characterized by prolonged blood circulation, delayed tumor accumulation, and high hepatic retention,^17,18^ whereas small-molecule and linear peptide tracers, despite faster pharmacokinetics, exhibit low tumor accumulation and rapid washout, decreasing imaging contrast and precluding their use in PD-L1-targeted radiotherapy.^19^ The design of high-affinity tracers remains particularly challenging due to the flat and hydrophobic binding surface of PD-L1 dimeric structure.^20^

Multicyclic peptides offer a promising solution. Covalent cross-linkers impose defined loop architectures that confer exceptional conformational stability, target selectivity, and resistance to proteolytic degradation.^21^ Disulfide-rich peptides (DRPs), defined by conserved cysteine patterns, represent a versatile scaffold encompassing natural, library-derived, and de novo-designed variants.^22^ Despite recent efforts in the design of DRPs that structurally mimic the PD-1/PD-L1 binding interface, their submicromolar affinities for PD-L1 have limited their application as PET tracers.^23^ The recent development of disulfide-directed multicyclic peptides (DDMPs) through the use of disulfide-directing biscysteine motifs has expanded accessible peptide structural space.^22,24,25^ Based on this concept, Cheng et al. reported a library-based DDMP platform enabling the discovery of novel PD-L1–binding multicyclic peptides with enhanced affinity.^26^

The discovery of CPXXC motifs (C: cysteine, P: proline, and X: any amino acid) has provided a powerful strategy for directing disulfide pairing and conformational folding in DRPs.^25^ As shown in Figure 1a, a DDMP library featuring two CPXXC motifs was designed to identify peptides that bind PD-L1. The peptide dmp1 was obtained from a dominant sequence enriched over three rounds of screening, followed by synthesis via Fmoc-SPPS and oxidative folding. Surface plasmon resonance (SPR) analysis indicated that dmp1 binds with PD-L1 with a *K*_D_ value of 2.14 μM. To improve the affinity of the DDMP library for PD-L1, a convergent secondary library and tertiary library were constructed, respectively. Following the third-round selection and next-generation sequencing (NGS) analysis, five lead variants (dmp2–6) with strong PD-L1 binding were identified from the enriched pool for synthesis and further examination. SPR results revealed that these selected peptides exhibit moderate nanomolar affinity for PD-L1.

**Figure 1:**
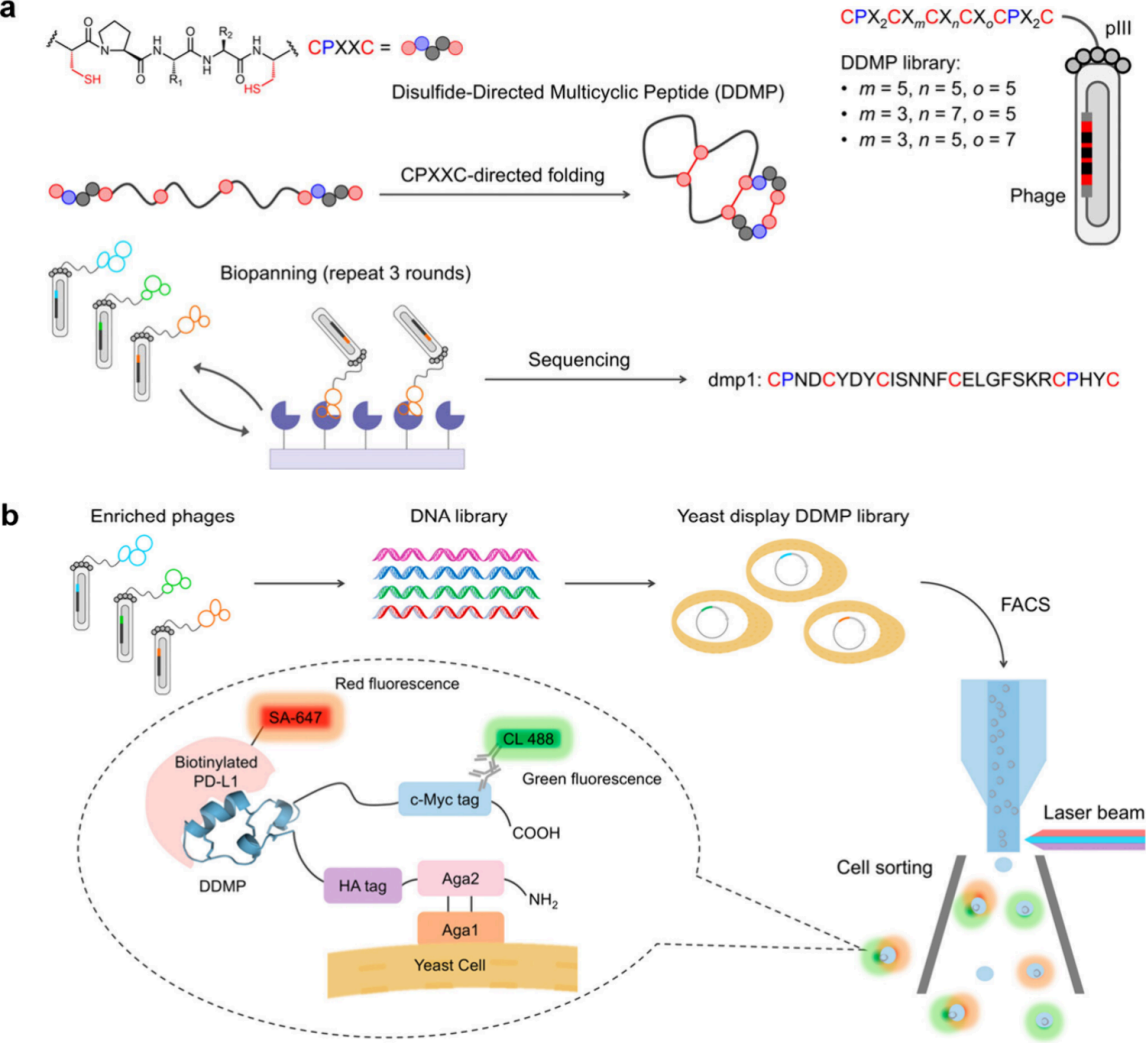
Identification of a PD-L1-targeting sequence (dmp1) from a Phage-Displayed DDMP Library (a). Schematic illustration of the yeast-displayed DDMP library construction using sequences obtained from prior phage display screenings (b). The figure was adapted from Cheng et al.^26^ Copyright 2025 American Chemical Society.

To further improve the binding affinity of these peptides, a yeast display library was constructed using sequences enriched from screening of a phage-displayed tertiary library (Figure 1b). Yeast cells displaying high-affinity PD-L1 binding peptides were isolated using fluorescence-activated cell sorting (FACS). After four rounds of cell sorting and selection, sequences dmp7 and dmp8 were predominantly enriched with *K*_D_ values of 45 and 54 nM, respectively. Using dmp7 as a template, a secondary yeast display library was generated via error-prone PCR (epPCR) to introduce random mutations and expand a diverse DNA mutant library. Following another four rounds of sorting, sequences dmp9 and dmp10 were identified via NGS analysis, exhibiting *K*_D_ values of 14 nM and 345 pM, respectively. SPR confirmed that dmp10 had a higher affinity than dmp9 due to a slower dissociation rate. Furthermore, dmp10 was highly selective for PD-L1 over PD-L2, a competitive ligand for the PD-1 receptor.

Confocal fluorescence imaging showed that fluorescein-labeled peptide (F-dmp10) bound specifically to PD-L1 with a *K*_D_ value of 6.5 nM. A SPR assay exhibited that dmp10 could block the PD-1/PD-L1 interaction effectively. This was also confirmed by its dose-dependent blockade of human PD-1 binding to cell-surface PD-L1 with an IC_50_ value of 17.1 nM. The X-ray crystal structure was employed to characterize the molecular interaction between dmp10 and PD-L1. The dmp10 peptide folds into a cyclic conformation and lies parallel to the PD-L1 β-sheet surface. It contains two α-helices (α1: Asp5–Arg11 and α2: Pro14–Leu18) and is stabilized by three disulfide bonds (Cys2–Cys28, Cys6–Cys24, and Cys10–Cys16) along with a hydrogen bond between the main-chain carbonyl of Ala4 and the side-chain NH of Arg23. The binding interface between PD-L1 and dmp10 features an array of noncovalent interactions. The dmp10/PD-L1 binding interface features a key T-shaped π-π stacking interaction between dmp10 Trp20 and PD-L1 Tyr123 within a hydrophobic cleft, complemented by an extensive hydrogen-bonding network and additional π-π stacking. These π-π stacking and hydrogen-bonding interactions confer excellent shape complementarity and high-affinity binding between dmp10 and PD-L1.

For *in vivo* imaging studies, dmp9 and dmp10 were conjugated to a DOTA chelator via a GSGSG linker to enable radiolabeling with ^68^Ga. [^68^Ga]­dmp9 and [^68^Ga]­dmp10 were prepared successfully with high efficiency (radiochemical yields >99%) and exhibited excellent *in vitro* and *in vivo* stability. PET/CT imaging of [^68^Ga]­dmp9 and [^68^Ga]­dmp10 was conducted in A375-PD-L1 tumor-bearing mice (Figure 2a). [^68^Ga]­WL12, a known single cyclic peptide targeting PD-L1, was included as a reference PD-L1 tracer for comparison.^27^ As shown in Figure 2a, [^68^Ga]­dmp10 exhibited the highest tumor accumulation at all time points, followed by [^68^Ga]­WL12 and [^68^Ga]­dmp9. The tumor accumulation of [^68^Ga]­dmp10 increased over time, reaching 13.27 ± 1.34%ID/g at 4 h post injection (p.i.). Both [^68^Ga]­dmp9 and [^68^Ga]­dmp10 demonstrated predominant kidney accumulation, reflecting their primary excretion through the renal system. Compared with [^68^Ga]­WL12, [^68^Ga]­dmp10 exhibited significantly reduced uptake in the liver (90%) and kidneys (40%). The combination of high tumor accumulation, prolonged tumor retention time, and low off-target accumulation makes [^68^Ga]­dmp10 an attractive agent for PD-L1-targeted radiotherapy.

**Figure 2:**
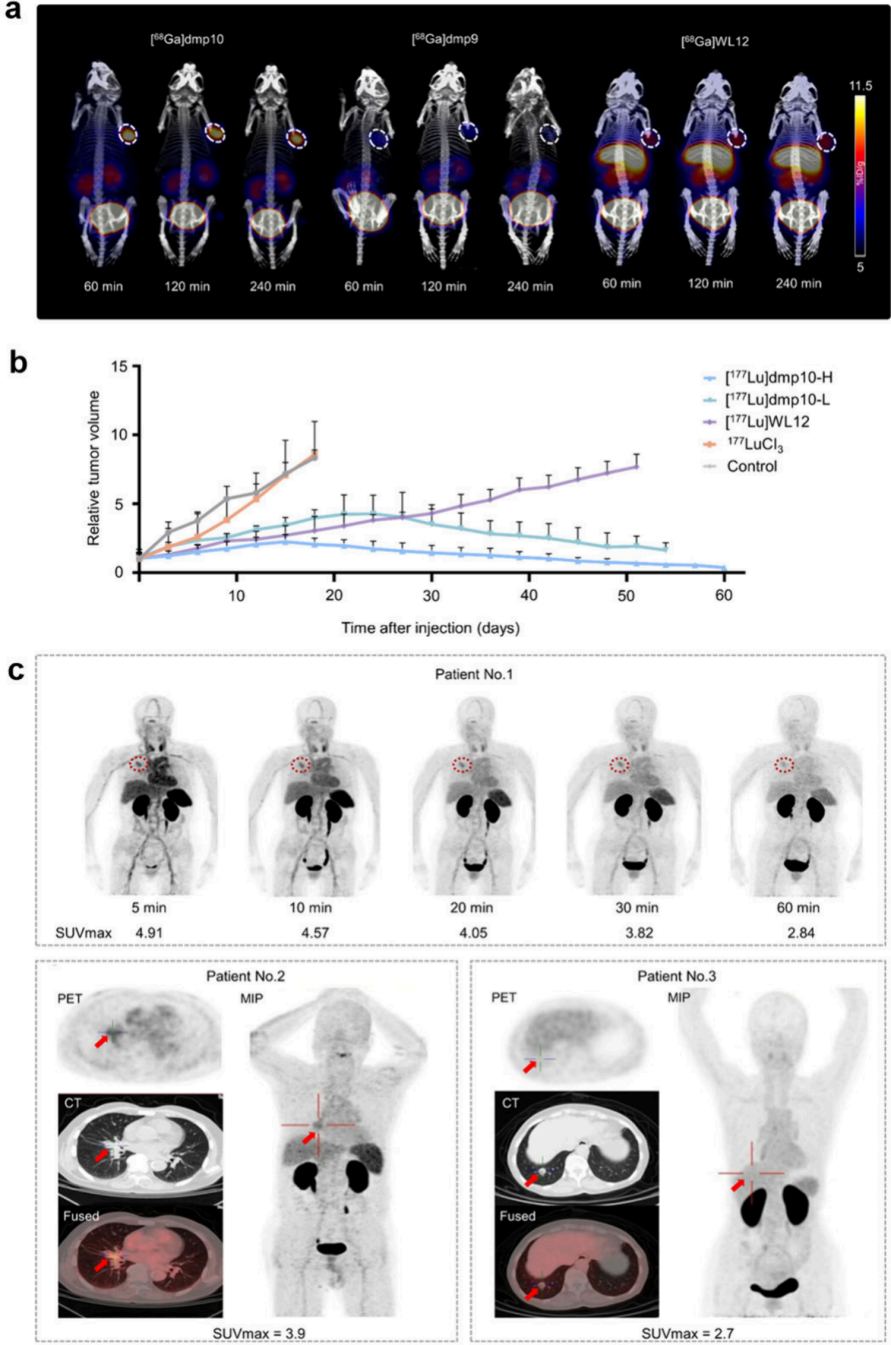
PET imaging of [^68^Ga]­dmp9, [^68^Ga]­dmp10 and [^68^Ga]­WL12 in A375-PD-L1 tumor-bearing mice (a). Therapeutic effect of [^177^Lu]­dmp10 with different doses and [^177^Lu]­WL12 in A375-PD-L1 tumor-bearing mice (b). PET/CT imaging of [^68^Ga]­dmp10 in Patient No. 1 with NSCLC, Patient No. 2 with NSCLC and Patient No. 3 with lung cancer metastasizing from colorectal cancer (c). The figure was adapted from Cheng et al.^26^ Copyright 2025 American Chemical Society.

To assess the therapeutic efficacy, dmp10 was labeled with ^177^Lu (Figure 2b). In A375-PD-L1 tumor-bearing mice, administration of [^177^Lu]­dmp10 resulted in marked tumor growth inhibition. After 18 days, the high-dose [^177^Lu]­dmp10 group achieved 75% reduction in tumor volume, compared with 53% in the low-dose [^177^Lu]­dmp10 group and 63% in the [^177^Lu]­WL12 reference group. A continued tumor regression was observed in [^177^Lu]­dmp10-treated mice between days 42 and 60 p.i., suggesting the potential to achieve complete tumor response. The [^177^Lu]­dmp10 group demonstrated a significantly higher survival rate, with 60% of mice surviving beyond 60 days, compared to only 20% in the [^177^Lu]­WL12 group. Toxicity assessment revealed no evidence of significant histopathological changes following treatment with [^177^Lu]­dmp10.

Given the promising preclinical data, [^68^Ga]­dmp10 was advanced into exploratory clinical evaluation in three patients. The PD-L1 expression in all three patients was confirmed by IHC. In patient 1, [^68^Ga]­dmp10 clearly delineated lung lesions with a PD-L1 tumor proportion score (TPS) of 10%, showing elevated uptake in right lung nodules (SUVmax = 2.84–4.91) that peaked at 30 min p.i., followed by a gradual decline over time. Patient 2 with NSCLC characterized by multiple nodular and cord-like opacities in the right lung lobe also showed evident uptake (SUVmax = 3.9 at 30 min p.i.) with a PD-L1 TPS of 2% (Figure 2c). In Patient 3 with colorectal cancer and lung metastases, the metastatic lesions demonstrated a positive [^68^Ga]­dmp10 signal, with a SUVmax of 2.7 and a PD-L1 TPS of 5%. These findings suggest that [^68^Ga]­dmp10 enables specific, noninvasive detection of PD-L1-expressing tumors, supporting its potential as a promising clinical tool for PD-L1-targeted cancer imaging.

## Future Outlook

In this study, dmp10, a multicyclic peptide that exhibits picomolar affinity for the target PD-L1 was developed through the discovery and engineering of DDMP. [^68^Ga]­dmp10 exhibited high tumor accumulation and prolonged tumor retention in xenograft mouse models. Preliminary clinical imaging data demonstrated that [^68^Ga]­dmp10 uptake correlated with PD-L1 expression in tumor tissues. However, given the small patient cohort, these findings warrant further validation in larger studies with systematic PD-L1 profiling to enable more effective patient selection, stratification, and therapeutic monitoring. While [^177^Lu]­dmp10 exhibited robust antitumor efficacy in preclinical studies using mouse tumor models with artificially high target expression, its therapeutic performance in human patients, whose tumors may exhibit heterogeneous or lower target levels, remains to be established. Notably, PD-L1 remains controversial as a useful biomarker because of its dynamic expression, induced by proinflammatory cytokines like γ-interferon during infection or inflammation.^28^ Moreover, clinical observations indicate that some PD-L1-negative patients still respond to PD-1/PD-L1 blockade.^29^ This phenomenon suggests that PD-L1 expression alone is not sufficient to predict therapeutic response, as other factors such as tumor-infiltrating lymphocytes, alternative immune checkpoints, and tumor mutational burden may also influence treatment outcomes. Given the spatial and temporal heterogeneity of PD-L1, molecular imaging provides a valuable tool to assess its dynamic distribution noninvasively. However, PD-L1 imaging should be interpreted in the context of comprehensive immunological profiling to more accurately guide patient selection and therapeutic monitoring. In all, the discovery of dmp10 expands the toolbox of PD-L1-targeted tracers and establishes a versatile strategy for developing high-affinity peptide ligands against protein–protein interaction.

## Abbreviations

PD-1: programmed cell death protein 1
PD-L1: programmed death-ligand 1
DDMP: disulfide-directed multicyclic peptide
ICB: immune checkpoint blockade
IHC: immunohistochemistry
PET: positron emission tomography
DRPs: disulfide-rich peptides
SPR: surface plasmon resonance
FACS: fluorescence-activated cell sorting
NGS: next-generation sequencing
NSCLC: nonsmall cell lung cancer
TPS: tumor proportion score
